# A Guide to Pain Assessment and Management in the Neonate

**DOI:** 10.1007/s40138-016-0089-y

**Published:** 2016-03-12

**Authors:** Norina Witt, Seth Coynor, Christopher Edwards, Hans Bradshaw

**Affiliations:** Department of Pediatrics, The University of Arizona, PO Box 245073, Tuscon, 85724 AZ USA; Departments of Pediatrics and Emergency Medicine, The University of Arizona, PO Box 245057, Tucson, 85724-5057 AZ USA; College of Pharmacy, The University of Arizona, Department of Pharmacy Services, Banner-University Medical Center, PO Box 210202, Tucson, AZ USA

**Keywords:** Neonatal, Pain management, Glucose and sucrose, Breastfeeding, Topical and local anesthetics, Acetaminophen, Morphine, Fentanyl, Ketamine

## Abstract

Newborn infants experience acute pain with various medical procedures. Evidence demonstrates that controlling pain in the newborn period is beneficial, improving physiologic, behavioral, and hormonal outcomes. Multiple validated scoring systems exist to assess pain in a neonate; however, there is no standardized or universal approach for pain management. Healthcare facilities should establish a neonatal pain control program. The first step is to minimize the total number of painful iatrogenic events when possible. If a procedure cannot be avoided, a tiered approach to manage pain using environmental, non-pharmacologic, and pharmacologic modalities is recommended. This systematic approach should decrease acute neonatal pain, poor outcomes, and provider and parent dissatisfaction.

## Introduction

 Newborn infants experience pain just as older children and adolescents experience pain; however, clinicians’ ability and approach to assessing and managing neonates is inadequate and controversial. Newborn infants experience acute measurable physiologic, behavioral, metabolic, and hormonal responses to pain [1••, 2]. They also experience long-term effects, including negative effects on neurologic and behavioral development. This is because the experience of pain occurs during a critical time of neurologic maturation [2]. In fact, preterm infants have demonstrated an exaggerated acute response to pain and worse behavioral and sensory long-term outcomes when compared to term neonates [3, 4]. Surmounting evidence demonstrates that controlling pain in the newborn period is beneficial, improving physiologic, behavioral, and hormonal outcomes [1••, 2].

So, why does it remain controversial? Assessing pain in a neonate is difficult as they are non-verbal and though multiple validated pain scoring systems exist; there is no standardized or universal approach to assessing neonatal pain. Additionally, there appears to be a lack of understanding of how neonates perceive pain and the resulting adverse sequelae that occur when pain remains untreated [2]. Thus, the use of pain control for neonates undergoing procedures is limited and inadequate [5, 6]. Further, until recently, there have been limited data on analgesia effectiveness and safety profiles.

In 2006, the American Academy of Pediatrics and the Canadian Pediatric Society published a policy stating that each healthcare facility should establish a neonatal pain control program aimed at routine assessment of pain, reduction in the number of painful procedures, and also reduction and prevention of acute pain from invasive procedures [7]. Most scientific literature regarding pain management and control is from the Neonatal Intensive Care Unit (NICU) where neonatal pain is commonly observed. In 2009, an Italian panel of expert neonatologists, Lago et al. established guidelines to assist clinicians with management of pain experienced by patients within the NICU [1••]. Neonates interface with clinicians outside of the NICU and thus potentially experience painful procedures within other venues as well. This includes the newborn nursery, outpatient clinics, the emergency department and the pediatric ward of the hospital. However, there have not yet been guidelines addressing pain assessment or management within these arenas. This review will explore the methods for assessing pain, the importance of establishing a universal approach for assessing pain as well as providing a tiered approach to managing pain in the neonate. Our goal is to improve pain scores (patient satisfaction), parental satisfaction, and provider satisfaction in all venues in which neonates are evaluated and treated.

## Pain Assessment–Pain Scales

Pain assessment in the non-verbal child and neonate can be a very challenging task in an already subjective process. There are pain scales used to assess pain; however, there are variations in the methods and scales used, and there is not a universal method to assess pain in this population. Objective measurements including heart rate, blood pressure, and salivary cortisol can be used, but most care providers usually rely on grimace, crying, and overall demeanor.

In addition to differences in pain scales, there are myriad of other factors that may influence perception and evaluation of pain. There is a demonstrable relationship between anxiety and pain perception in children as well as adults [8, 9••]. It is difficult, for example, to assess the impact of the often foreign and stressful nature of being in an emergency department or in an exam room. Another potentially powerful factor may be the dynamics of the parent–child relationship and the degree of stress the parent experiences when their child needs a painful procedure, which may be perceived by the child and lead to increased anxiety for the patient. Another often perhaps overlooked factor in the assessment of pain is the clinician’s skill and willingness to assess and interpret signs of pain. Studies demonstrate that there are significant differences between provider’s level of training and experience in the recognition of pain [10–12].

The Joint Commission standards for hospitalized patients make pain assessments mandatory for all patients [13]. The standard numeric 0–10 pain scale may be useful in verbal children; however, there are scales that have been validated for use in children as young as three for pain reporting [14–17]. The revised FACES pain scale, the Wong-Baker Faces scale, and the 10-cm visual analog scale are used in many healthcare settings to assess a pediatric patient’s pain [15, 17–22]. In addition to assessing pain by physiologic parameters in the neonatal population, there are multiple validated pain scales utilized by NICUs to assess pain. For example for term neonates: the neonatal infant pain scale (NIPS); neonatal facial coding system (NFCS); neonatal pain, agitation, and sedation scale (N-PASS); cry, required oxygen, increased vital signs, expression, sleeplessness scale (CRIES); COMFORT Scale; and Douleur Aigue Nouveau-ne (DAN) scoring system have all been described in the literature (see Table 1). The premature infant pain profile (PIPP) is a validated pain scoring system for preterm neonates [2, 17]. For infants, non-verbal young children, and in patients with cognitive impairment, the face, legs, activity, crying, and consolability (FLACC) scale or the revised FLACC scale can be used [23–30].

**Table 1:** Summary of neonatal pain scales [1]

Pain scale	What variables are included?	Type of pain	Notes
PIPP (premature infant pain profile)	Heart rate, oxygen saturation, facial actions	Procedural, postoperative	Reliable, valid, clinical utility is well established
NIPS (neonatal infant pain score)	Facial expression, crying, breathing patterns, arm and leg movements, arousal	Procedural	Reliable, valid
NFCS (neonatal facial coding system)	Facial actions	Procedural	Reliable, valid, clinical utility is well established, high degree of sensitivity to analgesia
N-PASS (neonatal pain, agitation and sedation scale)	Crying, irritability, facial expression, extremity tone, vital signs	Procedural, postoperative, mechanically ventilated patients	Reliable, valid. Includes sedation end of scale, does not distinguish pain from agitation
CRIES (cry, requires oxygen, increased vital signs, expression, sleeplessness)	Crying, facial expression, sleeplessness, requires oxygen to stay at >95 % saturation, increased vital signs	Postoperative	reliable, valid
COMFORT scale	Movement, calmness, facial tension, alertness, respiration rate, muscle tone, heart rate, blood pressure	Postoperative, critical care	Reliable, valid, clinical utility well established
DAN (Douleur Aiguë du Nouveau-né)	Facial expression, limb movements, vocal expression	Procedural	Reliable, valid

 It is important to note that there are no validated or widely studied scales to assess pain outside of the hospital setting. Studies regarding pain perception at home and familiar environments may provide more information regarding the role of the environment in a patient’s perception of pain. Furthermore, it may elicit information regarding the dynamic parent–child relationship and the complexity of a parent’s own perception of their child’s pain, their comfort with the hospital setting, and their own past experiences with pain and the hospital setting.

## Management of Neonatal Pain: A Tiered Approach

**Fig. 1:**
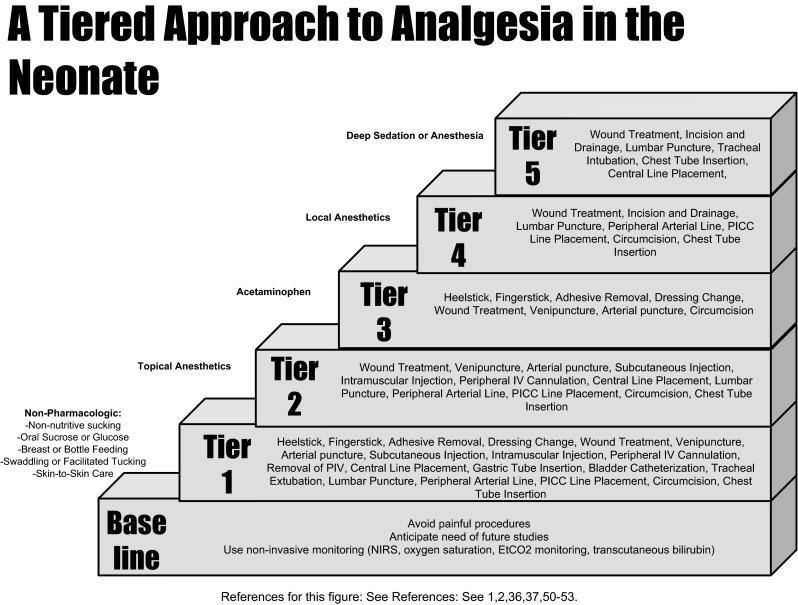
A tiered approach to analgesia in the neonate

Neonatal pain is best managed using a multi-directional approach which can be conceptualized in a tiered manner (see Fig. 1) and includes non-pharmacologic and pharmacologic modalities (see Table 2). The foundational basis for optimizing pain management in the neonatal population is aimed at reducing the total number of painful events [31]. This has been well established as a fundamental intervention employed in the NICU, where painful procedures are performed regularly. How can clinicians reduce the number of painful events? As noted in Fig. 1, at baseline, the approach should include avoiding unnecessary painful procedures. Further, clinicians should reduce the number of bedside interruptions and daily examinations, if possible. Additionally, clinicians can anticipate the need for future studies and, with thoughtful planning, can coordinate studies to minimize the frequency of blood draws [7]. Although a painful procedure within itself, painful blood draw frequency can further be reduced by insertion of arterial catheter or central venous line, if there is a need for more than three lab draws within 1 day [7]. Another way to reduce painful procedures is to use non-invasive monitoring when clinically relevant and when resources are available. These include near infrared spectroscopy (NIRS) monitoring, oxygen saturation monitoring, and obtaining bilirubin levels via transcutaneous bilirubinometer [1].

After laying the foundation for neonatal pain management, one can escalate therapy based on the degree of anticipated procedural pain, advancing through the appropriate tiers of therapy to achieve optimal analgesia including non-pharmacologic and pharmacologic modalities (see Table 2).

**Table 2:** Recommended neonatal and infant analgesic interventions and dosing

Analgesic	Type of procedure	Dosing	Other
Sucrose/glucose	Heel Lance Venipuncture	Oral: 20–30 % solution Multiple doses are more effective than single [38]	Optimal Dose has not yet been identified Decrease concentration in preterm neonates [1]
EMLA (2.5 % lidocaine +2.5 % prilocaine)	Venipuncture Circumcision PICC line insertion Lumbar puncture	Topical: 0.5–1 g covered with occlusive dressing × 45–60 min Max dose = 1 g	Not recommended for heel lance; more painful, longer procedure duration SE: common: skin irritation; Rare: methemoglobinemia [51, 53]
Acetaminophen	Heel lance Finger lance Adhesive removal Dressing change Wound treatment Venipuncture Circumcision	Oral: 10 mg/kg q6 h or 15 mg/kg q8 h [64] Rectal: 20–25 mg/kg IV: loading dose: 20 mg/kg, then maintenance with [56] TDD: 37–42 weeks = 50–60 mg/kg/day 1–3 months = 60–75 mg/kg/day	Neonates have slower clearance than older children [64] Rare SE: Hepatotoxicity, Renal Toxicity
Lidocaine injection	PICC line insertion Lumbar puncture Circumcision	SQ and IM: 3–5 mg/kg/dose of 0.5 % (5 mg/mL) or 1 % (10 mg/mL) [54]	Toxicity: arrhythmias, seizures Avoid combination with epinephrine in neonates-to minimize risk of arrhythmia and tissue necrosis J-tip/needle free have not been adequately studied [55]
Opiates	Wound treatment Incision and drainage Lumbar puncture Tracheal intubation Chest tube insertion	Morphine IV: 0.05–0.1 mg/kg/dose [75]	SE: Hypotension in preterm neonates [63, 65]
		Fentanyl IM/IV: 0.5–1 μg/kg/dose [75] Fentanyl intranasal: 1.5–2 μg/kg/dose [76]	SE: bradycardia, chest wall rigidity [70], but less hypotension, GI dysmotility and urinary retention than morphine [63, 65–70]
Ketamine	Procedural sedation	IM/IV: 0.5–2 mg/kg/dose	Bronchodilator: improves ventilation Minimal effects on respiratory drive, HR, BP Toxicity: >2 mg/kg/dose bradycardia [73]; >5 mg/kg/dose decreased BP [74]

*TDD* total daily dosing; *SE* side effects

## Tier 1: Non-pharmacologic Therapies

The first tier is aimed at employing non-pharmacologic therapies, which include oral sucrose or glucose, breast or bottle feeding, skin-to-skin care (aka Kangaroo Care), swaddling or facilitated tucking, non-nutritive sucking, and sensorial saturation. Of all non-pharmacologic therapies, the most robust literature is regarding the use of oral sucrose.

### Oral Sucrose

How do sugars affect pain? The proposed hypothesis is that glucose (and its alternative forms, such as sucrose) causes endogenous opioid release, through an unknown mechanism [32–34]. In a systematic review, Stevens et al. established that in the neonatal population, sucrose significantly reduces pain associated with procedures [35]. While the reported outcomes varied among the studies included in this meta-analysis, patients receiving sucrose were found to have significant reductions in behavioral and physiologic indicators of pain, as well as improvements on several different validated pain scores [35]. Specifically, measures of physiologic response, such as changes in heart rate, oxygen saturation, and vagal tone, were dampened when compared to placebo [35]. The use of sucrose in neonates, when compared to breast milk or pacifier use, has also been associated with a reduction in behavioral indicators of pain, such as crying and grimacing during painful procedures [35].

Glucose, in 20–30 % solutions, has similarly been studied as an effective alternative to sucrose therapy [36]. Glucose is recommended for venipuncture and heel lancing procedures, demonstrating a reduction in Premature Infant Pain Profile (PIPP) scores and the duration of crying in neonates [36]. In a recent systematic review, Bueno demonstrated that there has been no significant difference between the effectiveness of sucrose as compared to glucose [36]. Glucose has not yet been found to provide appropriate analgesia following more invasive or longer procedures, such as circumcision or eye examination for retinopathy of prematurity [36].

In studying their pharmacologic properties, glucose and sucrose have an ideal safety profile with limited side effects [36]. The recommended dosing ranges from 12 to 120 mg (24 % sucrose solution or 20–30 % glucose solution) [35–39]. It should be noted that the use of less-concentrated solutions is recommended in premature infants, as higher osmolar solutions (24–33 % sucrose/glucose) are thought to be associated with adverse outcomes [i.e., increased risk of necrotizing enterocolitis (NEC)] [1]. Providing sucrose in multiple doses, both before and after painful procedures, such as heel lancing, is more effective than a single dose [1]. It should also be noted that sucrose or glucose for analgesia is typically not effective after 3 months of age [2].

### Breastfeeding or Breast milk

If a patient is undergoing a single painful procedure, an alternative to use of sucrose or glucose or no intervention, is the use of breastfeeding or breast milk. Shah et al. demonstrated in a Cochrane review that breastfeeding appears to have an advantage for one-time painful procedures [40]. Neonates who were breastfed during heelstick procedures and venipunctures showed a significant decrease in the variability of physiologic response as compared to swaddling, holding by mother, placebo, pacifier use, or oral sucrose [40]. The physiologic parameters measured demonstrated a lower increase in heart rate, reduced duration of total crying time and also reduced the time to first cry [40]. Further, Shah demonstrated that there was significant reduction in standardized pain measures, such as PIPP scores, Douleur Aiguë du Nouveau-né (DAN) scores, neonatal infant pain scale (NIPS), and neonatal facial coding system (NFCS) scores [40]. Supplemental breast milk in lieu of breastfeeding was also analyzed in this systematic review, with variable results. In regard to physiologic parameters, the outcomes were favorable, as there was less of an increase in heart rate and decreased duration of total crying time [40]. Shah also notes a reduction in NFCS scores, as compared to the placebo group. However, when evaluated in comparison to NIPS and DAN, both validated scoring systems, there was no significant reduction in pain scores with supplemental breast milk. Is sucrose better? In Shah’s systematic review, sucrose, when compared to supplemental breast milk, demonstrated a greater reduction in physiologic parameters (reduced crying duration and lower heart rate variability) [40].

### Other Non-pharmacologic Therapies: Skin-to-Skin Contact, Positioning, and Non-nutritive Sucking

In the neonatal population, other environmental interventions have demonstrated effective reduction in pain, particularly when used as adjunctive therapy to sweet solutions and/or breastfeeding. Skin-to-Skin contact involves direct physical contact with the parent and baby and is also commonly known as Kangaroo Care for the close resemblance to marsupials’ approach to caring for their young. Skin-to-Skin contact is effective at reducing pain, in both physiologic parameters as well as reduction in PIPP scores [41]. Careful positioning such as swaddling a neonate and facilitated tucking, which involves manually flexing a neonate’s arms and the legs, both foster self-soothing behaviors and are effective at reducing pain in neonates [41]. Non-Nutritive Sucking has also been evaluated in preterm and term infants and is effective at reducing pain [42]. Non-nutritive sucking has been shown to have lower variability in heart rate and decreased crying time duration when compared to swaddling alone, no intervention, or rocking alone [43, 44]. Although these environmental measures reduce the pain associated with procedures, they are not as effective as when used in combination with other non-pharmacologic therapies.

### Combo of Non-pharmacologic Therapies Provides Synergistic Effect

The use of sucrose or glucose has the best effectiveness when used in combination with other non-pharmacologic therapies [36, 45]. Glucose or sucrose when used in combination with non-nutritive sucking reduces pain in neonates [36, 45]. Sensorial saturation, another method of pain reduction, involves multisensorial stimulation, including tactile, gustatory, auditory, and visual stimulation. Sensorial saturation used in combination with oral sucrose or glucose has been shown to even further reduce pain associated with minor painful procedures (i.e., lab draws) [46, 47]. For example, this would incorporate placing a sugary solution on the infant’s tongue and then providing a gentle facial massage while speaking calmly to the infant [46, 47]. Facilitated tucking is less effective than sucrose, however, when used in combination with sugary solutions has also demonstrated a synergistic effect [48]. Breastfeeding in combination with the use of glucose or sucrose has also demonstrated a reduction in pain compared to either individually [49]. Skin-to-Skin contact when used with glucose or sucrose reduces neonatal pain associated with minor procedures more than compared to either individually [50]. Thus, when feasible and appropriate resources are available, for single minor painful procedures, clinicians should aim to use combination of environmental and non-pharmacologic methods to achieve optimal analgesia.

## Tier 2–5: Pharmacologic Management

### Tier 2: Topical Anesthetics

Following the tiered approach to neonatal pain management, as noted in Fig. 1, Tier 2 involves the use of topical anesthetics. Multiple formulations of topical anesthetics are available for use in the pediatric population, including lidocaine 2.5 %/prilocaine 2.5 % (EMLA^®^), tetracaine cream 2 % (Ametop^®^, or Pontocaine^®^), liposomal lidocaine 4 % (LMX-4^®^) or liposomal lidocaine 5 % (LMX-5^®^), lidocaine 7 %/tetracaine 7 % (S-caine^®^), and benzocaine. However, most research in the neonatal population has been conducted with EMLA^®^. The use of Ametop or Pontocaine, LMX-4, LMX-5, and S-caine cannot be recommended for use in the neonatal population because safety and effectiveness have not yet been established [51, 52].

Topical benzocaine, an over-the-counter formulation for teething pain, should also be used with caution, as the high concentration of benzocaine (20 % = 200 mg/mL) can easily lead to overdose. Toxicity of these products can lead to multiple adverse effects, including methemoglobinemia and life-threatening arrhythmias, so caution should be used when choosing the appropriate topical anesthetic [51, 53]. The American Academy of Pediatrics does not recommend the use of topical anesthetics, specifically lidocaine or benzocaine, for teething pain because they have been associated with significant morbidity (seizures, respiratory depression, arrhythmias) and even death, but recommends using a teething ring (chilled but not frozen) and/or a gentle massage of the gums by the parent/caregiver. The FDA has also issued a black box warning against using over-the-counter topical anesthetics for teething pain [53].

Although many formulations of topical anesthetic are available, EMLA has been well established as effective in the neonatal population for reducing pain associated with minor procedures. EMLA is a eutectic mixture of lidocaine (2.5 %) and prilocaine (2.5 %) in a cream base. EMLA should be recommended for use in circumcision and venipuncture. With circumcisions, EMLA decreased facial grimacing, the total duration of crying time, heart rate variability, and oxygen desaturations when compared to placebo [54]. When performing venipuncture, it is recommended that clinicians use EMLA, as it reduces pain [51]. EMLA should also be used for analgesia with lumbar punctures, as it reduced heart rate variability, facial grimacing, and oxygen desaturations when compared to placebo [52]. Taddio notes equivocal results with pain reduction during peripherally inserted central catheter (PICC) line placement, but clinicians should still consider its use [51]. EMLA is not recommended for heelstick procedures because it is ineffective at reducing pain and may actually prolong the procedure [1, 51]. The recommended dose is 0.5 g to 1.0 g (maximum dose) applied to procedural site. This should be covered with an occlusive dressing for 45–60 min prior to procedure, which connotes necessity of non-emergent procedures [1]. EMLA has demonstrated effective safety profile; however, rare but serious side effects, such as methemoglobinemia, can occur. Methemoglobinemia is more likely in patients with underlying G6PD Deficiency or following excessive doses [51]. A common side effect of EMLA is transient skin irritation, which can occur with any of the topical anesthetics [51, 52].

### Tier 3: Acetaminophen

Acetaminophen is one of the most commonly used systemic medications in the neonatal population due to its well-established effectiveness at pain reduction as well as its favorable side effect profile. Acetaminophen is recommended for use in mildly to moderately painful procedures such as heelsticks, fingersticks, adhesive removal, dressing changes, wound treatment, venipuncture, arterial puncture, and circumcision [1, 51, 52, 55, 56]. There are various formulations, which have different dosing and clearance patterns, particularly in neonates. Oral acetaminophen should be dosed between 10 mg/kg every 6 h or 15 mg/kg every 8 h [56]. Neonates have slower clearance as compared to older children, so clinicians should be aware to dose less frequently [56]. There are limited data available for the use of IV acetaminophen in neonates, but recommended doses are loading dose of 20 mg/kg and then maintenance therapy administering 10 mg/kg every 6 h [57]. Total daily doses for neonates less than 1 month of age born between 37 and 42 weeks gestation is 50–60 mg/kg/day and is 60–75 mg/kg/day for infants 1–3 months postnatal age [58•]. Rectal acetaminophen should be dosed at 20 mg/kg every 6–8 h [57]. Acetaminophen in low doses is safe for use in neonates, but rare side effects should be noted including hepatic and renal toxicity [59, 60]. Acetaminophen is also helpful when used in combination with morphine. This combination has minimal adverse effects and reduces the total dose requirement of morphine to achieve equivalent pain reduction [61, 62•].

NSAIDS have well-established adverse effects in the neonatal population and are not recommended for use in the neonatal population [63]. Typically, use can begin for infants older than 6 months.

### Tier 4: Local Anesthetics

Traditional local anesthetics have been well established as effective in providing analgesia associated with painful procedures. This represents Tier 4 in the approach to neonatal analgesia. Lidocaine injections can safely reduce pain associated with PICC line, arterial line, central venous line placement, lumbar puncture, and circumcision. As noted with other measures, the use of lidocaine injections is most effective when used in combination with other interventions. For optimal pain relief with circumcision, sucrose use throughout the procedure and acetaminophen use for post-procedural pain was most effective [55]. In neonates, the recommended dose is 0.5 % (5 mg/mL) or 1 % (10 mg/mL) solution to a maximum dose of 3–5 mg/kg [55]. Again, as with EMLA, in higher doses, there is risk of arrhythmia and seizures when approaching toxic doses. Clinicians should avoid combination with epinephrine in neonates, to minimize the risk of arrhythmia and also tissue necrosis. It should also be noted that needle free formulations of lidocaine injections (J-Tip) have not been adequately studied in newborns [64].

### Tier 5: Deep Sedation

#### Opiates

The most commonly used opiates in the neonatal period are morphine and fentanyl. These systemic analgesics are typically reserved for moderately to severely painful procedures and should be adequately titrated accordingly. Such procedures include wound treatment, incision and drainage, lumbar puncture, tracheal intubation, chest tube insertion, and central line placement [1]. Much of the available evidence on the use of morphine and fentanyl in neonates has come from studies evaluating preterm infants within the NICU, who were typically mechanically ventilated. Thus, one should be careful when extrapolating this data to apply to a wider patient population including term neonates who are not mechanically ventilated. However, as noted before both morphine and fentanyl are commonly used for procedural pain control. In regard to side effect profile, hypotension has been associated with use of morphine in preterm infants, which was not found in term infants [65, 66].

Fentanyl provides rapid analgesia and has been well established as effective for pain reduction in tracheal intubation, chest tube insertion, incision and drainage, and postoperative procedural pain [67]. Fentanyl is an optimal choice in neonates because it has minimal hemodynamic effects, including less hypotension. It also has less GI dysmotility and urinary retention when compared with morphine [68–71]. However, bradycardia and chest wall rigidity are well-known side effects [72]. Further studies are needed to evaluate its safety and efficacy in use for one-time procedures. It should also be noted that alternative routes of administration including transmucosal, aerosolized, and inhaled fentanyl have demonstrated effectiveness similar to intravenous opioids [73, 77]. In mechanically ventilated neonates, fentanyl doses of 1–3 μg/kg are typically recommended to provide analgesia [72]. In non-intubated patients, lower doses, such as 0.5–1 μg/kg of IV fentanyl may be more appropriate, although further studies in this patient population are warranted. Naloxone is an effective reversal agent for opioid analgesics and should be readily available to reverse respiratory depression or other complications when opioids are used. Slow administration of fentanyl injections over 3–5 min decreases the potential for skeletal muscle/chest wall rigidity and associated impaired ventilation, respiratory distress or even respiratory arrest. Nondepolarizing skeletal muscle relaxants/paralytics such as rocuronium 1 mg/kg can be used to reverse chest wall rigidity but require endotrachial intubation for manual and/or mechanical ventilation, so prior preparation for potential intubation recommended with fentanyl usage.

#### Ketamine

Ketamine, an NMDA receptor antagonist, also known as a dissociative anesthetic, has come to favor more recently with regards to procedural sedation. The literature of its use in neonates is not as robust as literature supporting use in older pediatric and adult populations. Ketamine is ideal as it provides appropriate sedation, amnesia, and does not have hemodynamic instability as other well-established sedatives. Ketamine maintains respiratory drive, allows for bronchodilation, which improves ventilation and hemodynamic functioning, and has only minimal effects on heart rate and blood pressure [72]. Recommended dosing, as established in a subset of NICU neonates, is 1–2 mg/kg/dose. Doses greater than 2 mg/kg/dose are associated with reduction in heart rate [74]. The dose of 5 mg/kg has been associated with reduced blood pressure without impairing cardiac output [75].

#### Other Sedatives

Other sedative medications including anxiolytics such as midazolam, dexmedetomidine, and inhaled nitrous oxide have potential applications for use for painful procedures. However, not enough research has been conducted to establish their effectiveness at reducing pain in term neonates or potential adverse medication effects.

## Conclusion

 As we noted, newborns experience pain as measured by physiologic, behavioral, metabolic, and hormonal responses. They also experience long-term sequelae from pain including impaired neurologic and behavioral development. To date, there is no universal approach to neonatal pain assessment. Further, although there are guidelines for management of neonatal pain in the NICU, this approach has not translated to other venues where clinicians evaluate and treat neonates. We have reviewed validated pain scoring systems, such as PIPP, FLACC, NIPS, and DAN. Further, we recommend a tiered approach for the management of neonatal pain, including environmental, non-pharmacologic, and pharmacologic pain interventions. With a standardized approach to assessing and managing pain, we hope to improve acute neonatal pain, long-term neurologic and behavioral outcomes, as well as parent and provider satisfaction. Ultimately, further research is needed regarding efficacy, safety profiles, and satisfaction scoring to better achieve these goals.

## References

[1] *••* Lago P, Garetti E, Merazzi D, et al. Guidelines for procedural pain in the newborn. Acta Paediatr (Oslo, Norway: 1992). 2009;98(6):932–9. doi:10.1111/j.1651-2227.2009.01291.x. *A foundational systematic review of procedural pain prevention and treatment of NICU neonatal patients.* 10.1111/j.1651-2227.2009.01291.x PMC2688676 19484828

[2] Anand KJ, Aranda JV, Berde CB (2006). Summary proceedings from the neonatal pain-control group. Pediatrics.

[3] Frunau RVE, Anand JKS, Stevens BJ, McGrath PJ (2007). Long-term consequences of pain in human neonates. Pain in neonates.

[4] Peterson BS, Vohr B, Staib LH, Cannistraci CJ, DOlberg A, Schenier KC (2000). Regional brain volume abnormalities and long-term cognitive outcome in preterm infants. JAMA.

[5] Carbajal R, Rousset A, Danan C, Coquery S, Nolent P, Duerocq S (2008). Epidemiology and treatment of painful procedures in neonates in intensive care units. JAMA.

[6] Lago P, Guadagni AM, Merazzi D, Ancora G, Belleni CV, Cavazza A (2005). Pain management in the neonatal intensive care unit: a national survey in Italy. Pediatr Anesth.

[7] American Academy of Pediatrics Committee on Fetus and Newborn, American Academy of Pediatrics Section on Surgery, Canadian Paediatric Society Fetus and Newborn Committee (2006). Prevention and management of pain in the neonate: an update. Pediatrics.

[8] Koppal R, Ardash E, Uday A, Anilkumar G (2011). Comparison of the midazolam transnasal atomizer and oral midazolam for sedative premedication in paediatric cases. J Clin Diagn Res..

[9] *••* Fein JA, Zempsky WT, Cravero JP, The Committee on Pediatric Emergency Medicine and Section on Anesthesiology and Pain Medicine Pediatrics. Relief of pain and anxiety in pediatric patients in emergency medical systems. Pediatrics. 2012;130;e1391. doi:10.1542/peds.2012-2536. *Excellent guideline demonstrating a systematic approach to assessment, treatment and prevention of pain in pediatric patients within the Emergency Department.*

[10] Stevens BJ, Abbott LK, Yamada J, Harrison D, Stinson J, Taddio A, Barwick M, Latimer M, Scott SD, Rashotte J, Campbell F, Finley GA (2011). CIHR team in children’s pain. epidemiology and management of painful procedures in children in canadian hospitals. CMAJ.

[11] Karling M, Renström M, Ljungman G (2002). Acute and postoperative pain in children: a Swedish nationwide survey. Acta Paediatr.

[12] Carbajal R, Rousset A, Danan C, Coquery S, Nolent P, Ducrocq S, Saizou C, Lapillonne A, Granier M, Durand P, Lenclen R, Coursol A, Hubert P, de Saint Blanquat L, Boëlle PY, Annequin D, Cimerman P, Anand KJ, Bréart G (2008). Epidemiology and treatment of painful procedures in neonates in intensive care units. JAMA..

[13] Joint Commission on Accreditation of Healthcare Organizations (2001). Comprehensive accreditation manual for hospitals.

[14] Cohen LL, Lemanek K, Blount RL (2008). Evidence-based assessment of pediatric pain. J Pediatr Psychol..

[15] Jacob E, Wong D, Hockenberry MJ, Wilson D (2011). Pain assessment and management in children. Wong’s nursing care of infants and children.

[16] Beyer JE, Aradine CR (1986). Content validity of an instrument to measure young children’s perceptions of the intensity of their pain. J Pediatr Nurs.

[17] Scott J, Huskisson EC (1976). Graphic representation of pain. Pain.

[18] Hicks CL, von Baeyer CL, Spafford PA, vanKorlaar I, Goodenough B (2001). The faces pain scale-revised: toward a common metric in pediatric pain measurement. Pain.

[19] Belville RG, Seupaul RA (2005). Pain measurement in pediatric emergency care: a review of the faces pain scale-revised. Pediatr Emerg Care.

[20] Stinson JN, Kavanagh T, Yamada J, Gill N, Stevens B (2006). Systematic review of the psychometric properties, interpretability and feasibility of self-report pain intensity measures for use in clinical trials in children and adolescents. Pain.

[21] McGrath PJ, Walco GA, Turk DC, PedIMMPACT (2008). Core outcome domains and measures for pediatric acute and chronic/recurrent pain clinical trials: PedIMMPACT recommendations. J Pain..

[22] Lawrence J, Alcock D, McGrath P, Kay J, MacMurray SB, Dulberg C (1993). The development of a tool to assess neonatal pain. Neonatal Netw..

[23] Merkel SI, Voepel-Lewis T, Shayevitz JR, Malviya S (1997). The FLACC: a behavioral scale for scoring postoperative pain in young children. Pediatr Nurs..

[24] Malviya S, Voepel-Lewis T, Tait AR, Merkel S, Tremper K, Naughton N (2002). Depth of sedation in children undergoing computed tomography: validity and reliability of the University of Michigan Sedation Scale (UMSS). Br J Anaesth.

[25] Merkel S, Voepel-Lewis T, Malviya S (2002). Pain assessment in infants and young children: the FLACC scale. Am J Nurs.

[26] Munro HM, Walton SR, Malviya S (2002). Low-dose ketorolac improves analgesia and reduces morphine requirements following posterior spinal fusion in adolescents. Can J Anaesth.

[27] Riegger LQ, Voepel-Lewis T, Kulik TJ (2002). Albumin versus crystalloid prime solution for cardiopulmonary bypass in young children. Crit Care Med.

[28] Tait AR, Voepel-Lewis T, Robinson A, Malviya S (2002). Priorities for disclosure of the elements of informed consent for research: a comparison between parents and investigators. Paediatr Anaesth.

[29] Voepel-Lewis T, Merkel S, Tait AR, Trzcinka A, Malviya S (2002). The reliability and validity of the face, legs, activity, cry, consolability observational tool as a measure of pain in children with cognitive impairment. Anesth Analg.

[30] Malviya S, Vopel-Lewis T, Burke C (2006). The revised FLACC observational pain tool: improved reliability and validity for pain assessment in children with cognitive impairment. Paediatr Anaesth.

[31] Sharek PJ, Powers R, Koehn A, Anand KJ (2006). Evaluation and development of potentially better practices to improve pain management of neonates. Pediatrics.

[32] Blass E, Fitzgerald E, Kehoe P (1987). Interactions between sucrose, pain and isolation distress. Pharmacol Biochem Behav.

[33] Blass EM, Shah A (1995). Pain-reducing properties of sucrose in human newborns. Chem Sens.

[34] Ren K, Blass EM, Zhou QQ, Dubner R (1997). Suckling and sucrose ingestion suppress persistent hyperalgesia and spinal fos expression after forepaw inflammation in infant rats. Proc Natl Acad Sci USA.

[35] Stevens B, Yamada J, Ohlsson A (2010). Sucrose for analgesia in newborn infants undergoing painful procedures. Cochrane Database Syst Rev.

[36] Bueno M, Yamada J, Harrison D (2013). A systematic review and meta-analyses of nonsucrose sweet solutions for pain relief in neonates. Pain Res Manag.

[37] Taddio A, Shah V, Hancock R (2008). Effectiveness of sucrose analgesia in newborns undergoing painful medical procedures. CMAJ.

[38] Stevens B, Yamada J, Ohlsson A (2004). Sucrose for analgesia in newborn infants undergoing painful procedures. Cochrane Database Syst Rev.

[39] Johnston CC, Stremler R, Horton L, Friedman A (1999). Effect of repeated doses of sucrose during heel stick procedure in preterm neonates. Biol Neonate.

[40] Shah PS, Aliwalas LI, Shah V (2006). Breastfeeding or breast milk for procedural pain in neonates. Cochrane Database Syst Rev..

[41] Johnston C, Campbell-Yeo M, Fernandes A, Inglis D, Streiner D, Zee R (2014). Skin-to-skin care for procedural pain in neonates. Cochrane Database Syst Rev.

[42] Pillai Riddell RR, Racine NM, Turcotte K (2011). Non-pharmacological management of infant and young child procedural pain. Cochrane Database Syst Rev.

[43] Campos RG (1989). Soothing pain-elicited distress in infants with swaddling and pacifiers. Child Dev.

[44] Campos RG (1994). Rocking and pacifiers: two comforting interventions for heelstick pain. Res Nurs Health.

[45] Bellieni CV, Bagnoli F, Perrone S (2002). Effect of multisensory stimulation on analgesia in term neonates. A randomized controlled trial. Pediatr Res.

[46] Bellieni CV, Buonocore G, Nenci A, Franci N, Cordelli DM, Bagnoli F (2001). Sensorial Saturation: an effective analgesic tool for heel-prick in preterm infants. Biol Neonate.

[47] Bellieni T, Coccina B (2012). Sensorial saturation for infants’ pain. J Matern Fetal Neonatal Med.

[48] Cignacco EL, Sellam G, Stoffel L (2012). Oral sucrose and “facilitated tucking” for repeated pain relief in preterms: a randomized controlled trial. Pediatrics.

[49] Gradin M, Finnstrom O, Schollin J (2004). Feeding and oral glucose-additive effects on pain reduction in newborns. Early Hum Dev.

[50] Chermont AG, Falcao LF, de Souza Silva EH, de Cassia Xavier Balda R, Guinsburg R (2009). Skin-to-skin contact and/or oral 25% dextrose for procedural pain relief for term newborn infants. Pediatrics.

[51] Taddio A, Ohlsson A, Einarson TR (1998). A systematic review of lidocaine-prilocaine cream (EMLA) in the treatment of acute pain in neonates. Pediatrics.

[52] Kaur G, Gupta P, Kumar A (2003). A randomized trial of eutectic mixture of local anesthetics during lumbar puncture in newborns. Arch Pediatr Adolesc Med.

[53] US Food and Drug Administration. FDA drug safety communication: reports of a rare, but serious and potentially fatal adverse effect with the use of over-the-counter (OTC) benzocaine gels and liquids applies to the gums or mouth.

[54] Taddio A, Ohlsson K, Ohlsson A (2000). Lidocaine-prilocaine cream for analgesia during circumcision in newborn boys. Cochrane Database Syst Rev.

[55] Roman- Rodriguez CF, Toussaint T, Sherlock DJ (2014). Pre-emptive penile ring block with sucrose analgesia reduces pain response to neonatal circumcision. Urology.

[56] Shah V, Taddio A, Ohlsson A (1998). Randomised controlled trial of paracetamol for heel prick pain in neonates. Arch Dis Child Fetal Neonatal Ed.

[57] Allegaert K, Palmer GM, Anderson BJ (2011). The pharmacokinetics of intravenous paracetamol in neonates: size matters most. Arch Dis Child.

[58] • Cuzzolin L, Antonucci R, Fanos V. Paracetamol (acetaminophen efficacy and safety in the newborn. Curr Drug Metab. 2013;14:178. *Quantifies the safe total daily dose of acetaminophen in the pre*-*term and term infants. Also, for infants 1*–*3* *months of age, which had previously not been established.* 22935063

[59] Trugo R, Anand KJ (1989). Management of pain in the postoperative neonate. Clin Perinatol.

[60] Anderson BJ, Wollard GA, Holford NH (2000). A model for size and age changes in the pharmacokinetics of paracetamol in neonates, infants and children. Br J Clin Pharmacol.

[61] Ceelie I, de Wildt SN, van Dijk M (2013). Effect of intravenous paracetamol on postoperative morphine requirements in neonates and infants undergoing major noncardiac surgery: a randomized controlled trial. JAMA.

[62] • Anand KJ. Pain panacea for opiophobia in infants? JAMA. 2013;309:183. *Reviews the above article by Ceelie (reference 62). Noting pros of the study, which included careful study design and well*-*matched study groups. Cons included small sample size, single*-*center study site, and lack of safety data. Further, he points out that there was only a brief duration of exposure to morphine 48*–*72 hours, which is the window in which opioid induced hyperalgesia could have increased analgesic requirements. Notes that there is a possibility of synergism between morphine and acetaminophen. Notes that infants are potentially less susceptible to hepatic toxicity from oral or rectal acetaminophen because of their slightly delayed maturation of cytochrome P450 enzyme and higher glutathione stores.* 10.1001/jama.2012.208359 23299611

[63] Bhatt-Mehta V, Rosen DA (1991). Management of acute pain in children. Clin Pharm.

[64] Zempsky WT, Bean-Lijewski J, Kauffman RE (2008). Needle-free powder lidocaine delivery system provides rapid effective analgesia for venipuncture or cannulation pain in children: randomized, double-blind comparison of venipuncture and venous cannulation pain after fast-onset needle free powder lidocaine or placebo treatment trial. Pediatrics.

[65] Carbajal R, Lenclen R, Jugie M (2005). Morphine does not provide adequate analgesia for acute procedural pain among preterm neonates. Pediatrics.

[66] Taddio A, Lee C, Yip A (2006). Intravenous morphine and topical tetracaine for treatment of pain in neonates undergoing central line placement. JAMA.

[67] Pereira e Silva Y, Gomez RS, Marcatto Jde O (2007). Morphine versus remifentanil for intubating neonates. Arch Dis Child Fetal Neonatal Ed.

[68] Saarenmaa E, Huttunen P, Leppaeluoto J (1999). Advantages of fentanyl over morphine in analgesia for ventilated newborn infants after birth: a randomized trial. J Pediatr.

[69] Ionides SP, Weiss MG, Angelopoulos M (1994). Plasma beta-endorphin concentrations and analgesia-muscle relaxation in the newborn infant supported by mechanical ventilation. J. Pediatr.

[70] Franck LS, Vilardi J, Durand D, Powers R (1998). Opioid withdrawal in neonates after continuous morphine or fentanyl during extracorporeal membrane oxygenation. Am J Crit Care.

[71] Anand KJ, Willson DF, Berger J (2010). Tolerance and withdrawal from prolonged opioid use in critically ill children. Pediatrics.

[72] Hall RW, Shbarou RM (2009). Drugs of choice for sedation and analgesia in the neonatal ICU. Clin Perinatol.

[73] Litman RS, Berkowitz RJ, Ward DS (1996). Levels of consciousness and ventilatory parameters in young children during sedation with oral midazolam and nitrous oxide. Arch Pediatr Adolesc Med.

[74] Saeerenmaa E, Neuvonen PJ, Huttunen P, Fellman V (2001). Ketamine for procedural pain relief in newoborn infants. Arch Dis Child Fetal Neonatal Ed.

[75] Betremieux P, Carre P, Pladys P (1993). Doppler ultrasound assessment of the effects of ketamine on neonatal cerebral circulation. Dev Pharmacol Ther.

[76] Anand KJ, International Evidence-Based Group for Neonatal Pain (2001). Consensus statement for the prevention and management of pain in the newborn. Arch Pediatr Adolesc Med.

[77] Harlos MS (2013). Intranasal fentanyl in the palliative care of newborns and infants. J Pain Symptom Manag.

